# Clinical, hematological and biochemical alterations in hamster (*Mesocricetus auratus*) experimentally infected with *Leishmania infantum* through different routes of inoculation

**DOI:** 10.1186/s13071-016-1464-y

**Published:** 2016-03-31

**Authors:** Nádia das Dores Moreira, Juliana Vitoriano-Souza, Bruno Mendes Roatt, Paula Melo de Abreu Vieira, Wendel Coura-Vital, Jamille Mirelle de Oliveira Cardoso, Mariana Trevisan Rezende, Henrique Gama Ker, Rodolfo Cordeiro Giunchetti, Claudia Martins Carneiro, Alexandre Barbosa Reis

**Affiliations:** Laboratório de Imunopatologia, Núcleo de Pesquisas em Ciências Biológicas/NUPEB, Universidade Federal de Ouro Preto, Ouro Preto, Minas Gerais Brasil; Laboratório de Pesquisas Clínicas, Departamento de Análises Clínicas, Escola de Farmácia, Universidade Federal de Ouro Preto, Ouro Preto, Minas Gerais Brasil; Laboratório de Morfopatologia, Departamento de Ciências Biológicas, Universidade Federal de Ouro Preto, Ouro Preto, Minas Gerais Brasil; Laboratório de Biologia das Interações Celulares, Departamento de Morfologia, Universidade Federal de Minas Gerais, Belo Horizonte, Minas Gerais Brasil

**Keywords:** Hamster, *Mesocricetus auratus*, *Leishmania infantum*, Experimental infection, Hematological and biochemical alterations

## Abstract

**Background:**

Leishmaniasis remains among the most important parasitic diseases in the developing world and visceral leishmaniasis (VL) is the most fatal. The hamster *Mesocricetus auratus* is a susceptible model for the characterization of the disease, since infection of hamsters with *L. infantum* reproduces the clinical and pathological features of human VL. In this context, it provides a unique opportunity to study VL in its active form. The main goal of this study was to evaluate the clinical, biochemical, and hematological changes in male hamsters infected through different routes and strains of *L. infantum.*

**Methods:**

In the current study, hamsters (*Mesocricetus auratus*) were infected with the *L. infantum* strains (WHO/MHOM/BR/74/PP75 and MCAN/BR/2008/OP46) by intradermal, intraperitoneal and intracardiac routes. The animals were monitored for a nine  month follow-up period.

**Results:**

The hamsters showed clinical signs similar to those observed in classical canine and human symptomatic VL, including splenomegaly, severe weight loss, anemia, and leucopenia. Therefore the OP46 strain was more infective, clinical signs were more frequent and more exacerbated in IC group with 80 to 100 % of the animals showing splenomegaly, in the last month infection. Additionally, desquamation, hair loss and external mucocutaneous lesions and ulcers localized in the snout, accompanied by swelling of the paws in all animals, were observed. Consequently, the animals presented severe weight loss/cachexia, hunched posture, an inability to eat or drink, and non-responsiveness to external stimuli. Furthermore, regardless of strain, route of inoculum and time assessed, the animals showed renal and hepatic alterations, with increased serum levels of urea and creatinine as well as elevated serum levels of aspartate aminotransferase and alanine aminotransferase.

**Conclusions:**

These results strongly suggest that the inoculation through the intracardiac route resulted in a higher severity among infections, especially in the sixth and ninth month after infection via intracardiac, exhibited clinical manifestations and biochemical/hematological findings similar to human visceral leishmaniasis. Therefore, we suggest that this route must be preferentially used in experimental infections for pathogenesis studies of VL in the hamster model.

## Background

Human visceral leishmaniasis (HVL) is a severe disease, with a high degree of mortality if not diagnosed and treated appropriately. Visceral leishmaniasis (VL) has an estimated incidence of 500,000 new cases arising each year worldwide and can be accompanied by the appearance of various typical clinical signs such as chronic fever, hepatosplenomegaly, weight loss/cachexia, progressive anemia, pancytopenia and hypergammaglobulinemia [1–3]. The typical outcome of symptomatic clinical VL is critically influenced by the host’s immune response, and systemic infection is characterized by the spread of the parasites mainly to the spleen, liver, lymph nodes and bone marrow [4–8]. Progress in developing new prophylactic and therapeutic strategies depends on animal models that can reproduce the course of the human disease to allow an understanding of the infection and its evolution [7, 9]. Nonetheless, in animals susceptible to infection by *Leishmania*, the outcome of experimental infection depends not only on host immunity but also on a combination of factors, such as inoculated species, strain virulence, nature of the inoculum, number of parasites and route of inoculation [7, 10].

Different species of experimental animals, such as mice and hamsters, have been used for studying HVL [5–7]. Mice are genetically resistant or susceptible to infection but even susceptible mice can contain the infection and prevent overt disease [11]. Initially, parasite growth is observed in the liver and spleen, but within 4–5 weeks of the infection, the liver parasitism is resolved by a Th1-dependent granulomatous response [4, 12, 13]. Thereafter, the parasite persists as a chronic infection in the spleen, with gradual destruction of the organ architecture [5, 14–16]. Several other experimental animal models of VL have been developed through the years [7, 17–23]. Of these, the hamster (*Mesocricetus auratus*) represents a good model for VL as it has a unique susceptibility to a variety of intracellular pathogens [14, 23]. Moreover, it develops the majority of immunopathological alterations characteristic of the human disease, with growth of parasites in the bone marrow, spleen and liver; clinical and hematological symptoms such as hepatosplenomegaly, depressed lymphocyte proliferation, anemia, leucopenia and glomerulonephritis; death by 9–10 weeks after visceral infection [7, 14]. This model has been extensively used for studies on disease pathogenesis and immunosuppression [14, 24–26] and to test efficacy of drugs and vaccines [26–32].

The susceptibility of hamsters to infection by *Leishmania* spp. depends on a number of host factors (e.g. age and sex) that plays an important role in modulating the immune system [33, 34]. Male hamsters infected at a juvenile stage have been shown to be more susceptible to infection and to have higher and more severe lesions and greater parasite burden in lymph nodes compared with females [35]. After challenge, male hamsters showed decreased weight gain and higher mortality rates at any given time point than female or control male hamsters. Body weight has been shown to be a relevant determinant of the clinical outcome of the infection in hamsters with VL [36].

In a paper published recently [7], we noticed that the route of infection and the type of strain influence the humoral response and parasite load in the liver and spleen of hamsters experimentally infected with *L. infantum.* In addition, we have shown  the importance of PCR to determine the parasitism in a hamster model, given intense use of this model in the last decade to test VL drugs and vaccines [7]. However, it is not clear, how the parasite alters the clinical and laboratory parameters in hamsters experimentally infected with *L. infantum* and how these alterations contribute to the pathology associated with VL. In this study the main goal was to investigate the clinical and laboratory changes of the animals inoculated with *L. infantum* strains (MHOM/BR/74/PP75 and MCAN/BR/2008/OP46) by (intradermal [ID], intraperitoneal [IP], and intracardiac [IC]) routes.

## Methods

### Ethical approval

This study was reviewed and approved by the Institutional Animal Ethics Committee (approval ID number 2009/09). The experimental procedures were carried out in strict accordance with the recommendations of the Brazilian regulations relating to experimental biology and medicine.

### Animals

One-month-old male Syrian golden hamsters (*Mesocricetus auratus*) were obtained from the Central Animal Facility at the Universidade Federal de Ouro Preto (CCA/UFOP). The animals were housed in appropriate plastic cages and fed with standard rodent food pellet and water *ad libitum*.

### *Leishmania* parasites and experimental infection

The *L. infantum* strains MHOM/BR/74/PP75 and MCAN/BR/2008/OP46 were used in experimental infection of hamsters. The MCAN/BR/2008/OP46 strain was isolated from a symptomatic naturally infected dog provided by the Center of Zoonosis Control (CCZ), Governador Valadares, Brazil. The parasite growth, inoculums and infection were carried out  as previously described by Moreira et al.[7].

### Experimental groups, follow-up, and collection of samples

Two groups of 120 hamsters each were infected with *L. infantum* (WHO/MHOM/BR/74/PP75 and MCAN/BR/2008/OP46) strains, respectively. Hamsters were inoculated with 1 × 10^7^ promastigotes of *L. infantum* in the stationary growth phase by intradermal, (*n* = 40), intraperitoneal (*n* = 40), and intracardiac (*n* = 40), routes. Eighty uninfected hamsters were used as a control group (C, *n* = 80). The animals were monitored for a nine  month follow-up period. The promastigotes of *L. infantum* were concentrated in a final volume that depended on the inoculation route to be used. For the ID route, 20 μL of inoculum was injected into the right ear of each animal, while 500 μL was used for IP inoculation. IC inoculation required a final volume of 200 μL in animals that were anesthetized with 2.5 % sodium pentobarbital at a dose of 10 mg/kg intraperitoneally.

Blood samples were collected by cardiac punction from each of the 10 hamsters per group at 1, 3, 6 and 9 months after inoculation. One blood sample from each animal was centrifuged to obtain serum for biochemical examinations, and a second sample was collected and transferred to a tube containing EDTA (Sigma Chemical Co) as the anticoagulant for measurement of hematological parameters. The hamsters were subsequently euthanized, and the liver and spleen were collected aseptically and weighed, and lesions were analyzed based on color, size, shape, surface appearance and consistency.

### Clinical evaluation

The hamsters were monitored for appearance, activity, swelling, pain, desquamation, hair loss and ulceration. Animals were weighed using a calibrated balance (analytical balance FA-2104 N Bioprecisa) before *L. infantum* infection and on the day of euthanasia (1, 3, 6 and 9 months after inoculation). The critical point for this study was reached at nine months after infection when hamsters exhibited any of the following criteria: severe weight loss, inability to eat or drink, or non responsiveness to external stimuli.

### Blood sample collection for hematological and biochemical analysis

Blood was collected by cardiac puncture after administration of anesthetic (sodium thiopental 2.5 % at dose of 10 mg/kg intraperitoneally) and transferred to tubes containing EDTA (Sigma Chemical Co) as the anticoagulant. The absolute counts for leukocytes, erythrocytes, hematocrit, hemoglobin, and platelets in each sample was obtained using an auto Hematology Analyzer (Mindray BC-2800Vet, Hamburg, Germany). The leukocyte count was determined by the number of leukocytes per cubic millimeter. The differential cell count was performed in the blood smears stained by Giemsa and cell counts were performed by a blood cell counter to determine the absolute number of cells, using an optical microscopy.

Blood for biochemical analysis was collected in glass tubes without anticoagulant and then centrifuged to collect the serum. The biochemical evaluations consisted of the following tests: determination of urea and creatinine to gauge renal function and the measurement of alanine aminotransferase (ALT) and aspartate aminotransferase (AST) as liver function tests. For these analyses, Automatic Biochemical (CELM SBA-200, Barueri, SP, and Brazil) and Commercial Labtest kits (Labtest Diagnostica SA, Lagoa Santa, MG, and Brazil) were employed following the methods described by the manufacturers.

### Parasite load

The parasite load was detected by quantitative real-time PCR methods as described elsewhere by Moreira et al. [7]. Briefly, total genomic DNA was extracted from approximately 20 mg of tissue (spleen and liver). For spleen samples, extraction with Wizard TM Genomic DNA Purification Kit (Promega H, Madison, WI, USA) was used following manufacturer’s recommendations. To obtain the DNA of the liver samples the CTAB method was used as previously described by Moreira et al. [7]. The concentration of DNA obtained from tissues was determined with a spectrophotometer (NanoVue Plus, GE Healthcare Products, Piscataway, NJ, USA). In order to quantify parasite burdens, we used the following primers: forward: 5′ TGT CGC TTG CAG ACC AGA TG 3′, and reverse: 5′GCA TCG CAG GTG TGA GCA C 3′ that amplified a 90-bp fragment of a single-copy of the DNA polymerase gene of *L. infantum* (GenBank accession number AF009147). PCR was carried out in a final volume of 25 μL containing 200 nM forward and reverse primers, 1× SYBER GREEN reaction master mix (Applied Biosystems, USA), and 5 μL of template DNA. Standard curves were prepared for each run using known quantities of pGEM®-T plasmids (Promega, USA) containing inserts of interest. The results were expressed as the number of amastigotes in 20 ng of DNA of tissue (spleen and liver).

### Statistical analysis

All analyses were conducted using Prism 5.0 software package (Prism Software, Irvine, CA, USA). Normality of the data was established using the Kolmogorov-Smirnoff test. Differences between experimental groups were performed using one-way ANOVA followed by Tukey’s test. Spearman’s rank correlation was also performed to investigate associations between parasite load and relative weight of organs. Differences with *P* < 0.05 were considered significant.

## Results

### The clinical pathological signs and evolution of hamster mimics the classic canine and human symptomatic VL

**Fig. 1:**
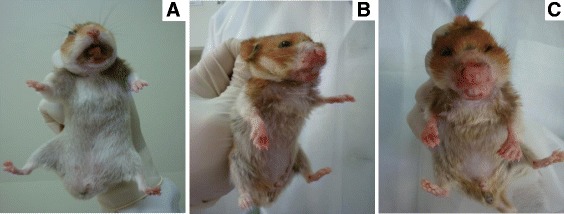
Macroscopic changes observed in hamsters 9 months after infection with the OP46 strain. In **a** normal hamster, **b**-**c** severe cachexia, hair loss, swelling in the paws and mucocutaneous lesions (snout)

To evaluate the progression of infection, clinical signs were analyzed at 1, 3, 6 and 9 months after inoculation with PP75 and OP46 *L. infantum* strains via three different routes (ID, IP and IC). Clinical signs suggestive of VL were observed in all groups and were generally more evident at 6 and 9 months after experimental infection for both strains. Animals inoculated with the PP75 strain via the IP route showed clinical signs earlier; however, the symptoms disappeared by the ninth month of monitoring. The animals infected with PP75 strain via the ID and IC routes showed clinical signs after 6 months, which were more severe at 9 months in hamsters inoculated via the IC route. Hamsters infected with the OP46 strain showed clinical signs suggestive of VL (splenomegaly and ascites) from 3 months after infection regardless of route. Overall, the OP46 strain was associated with more severe clinical signs than PP75 during the monitoring period for all three routes (Table 1). Interestingly, 9 months after IC infection with the OP46 strain, we found desquamation, hair loss, and external mucocutaneous lesions and ulcers localized in the snout, accompanied by edema of the paws in all animals. Furthermore, the animals exhibited severe weight loss, hunched posture, an inability to eat or drink and non-responsiveness to external stimuli (Fig. 1).

**Table 1:** Clinicopathological analysis of hamsters experimentally infected with *L. infantum*. Absolute (n) and percentage (%)

Time/infection (months)	Clinical signs	Experimental groups, n (%)					
		MHOM/BR/74/PP75 strain			MCAN/BR/2008/OP46 strain		
		ID	IP	IC	ID	IP	IC
1	–	–	–	–	–	–	–
3	Splenomegaly	–	1/10 (10)	–	–	1/10 (10)	8/10 (80)
	Ascites	–	1/10 (10)	–	6/10 (60)	1/10 (10)	–
6	Splenomegaly	–	–	2/10 (20)	1/10 (10)	7/10 (70)	10/10 (100)
	Hepatomegaly	1/10 (10)	–	–	–	–	1/10 (10)
	Ascites	4/10 (40)	4/10 (40)	3/10 (30)	4/10 (40)	1/10 (10)	6/10 (60)
	Emaciation^a^	–	–	–	1/10 (10)	–	9/10 (90)
9	Splenomegaly	1/10 (10)	–	7/9 (77)	1/10 (10)	7/8 (87)	6/6 (100)
	Hepatomegaly	–	–	3/9 (33)	1/10 (10)	–	1/6 (16)
	Ascites	1/10 (10)	–	1/9 (11)	–	3/8 (37)	6/6 (100)
	Emaciation^b^	–	–	2/9 (22)	–	–	6/6 (100)
	Skin lesion	–	–	–	–	–	6/6 (100)
	Swelling of the paw	–	–	–	–	–	6/6 (100)

Animals were infected with PP75 and OP46 strains of *L. infantum* by different routes of inoculation: intradermal (ID), intraperitoneal (IP), and intracardiac (IC)

^a^Weight loss with preservation of the general condition of the animal

^b^Accentuated/cachexia weight loss (observed only in the group inoculated by the IC route)

**Fig. 2:**
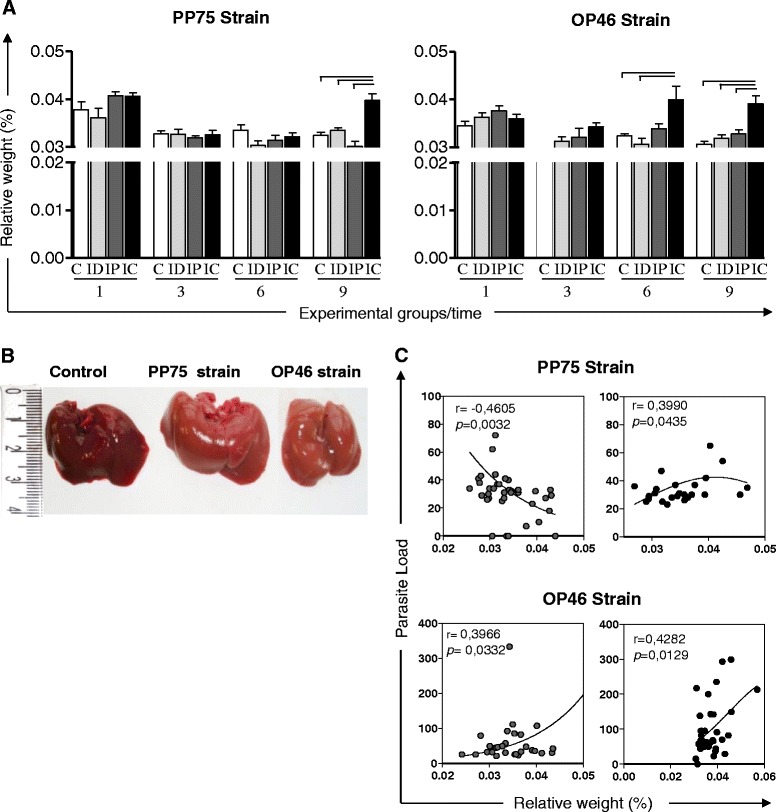
Relative weight of the liver of uninfected hamsters as a control group (C, *n* = 10 animals/time; white) and hamsters experimentally infected with *L. infantum* (PP75 or OP46 strain) by routes of inoculation: intradermal (ID, *n* = 10 animals/time; light gray), intraperitoneal (IP, *n* = 10 animals/time; dark gray), or intracardiac (IC, *n* = 10 animals/time; black) after 1, 3, 6, and 9 months of infection. The results are expressed as mean ± standard deviation. Significant differences (*P* < 0.05) between infection with the different routes of inoculation are represented by the connected lines (**a**). Macroscopic changes in the liver of uninfected hamsters as a control group (C; white) and in hamsters experimentally infected with *L. infantum* (PP75 or OP46) by the IC route of inoculation at 9 months of infection. The liver of control hamsters with a normal appearance; liver of hamsters infected with strains PP75 and OP46 appeared friable; changed color from red to pale yellowish brown (**b**). Correlation between parasite load and relative weight of the liver in hamsters experimentally infected with *L. infantum* (PP75 or OP46 strain) by routes of inoculation: intraperitoneal (IP; dark gray) or intracardiac (IC; black) after 1, 3, 6 and 9 months of infection. Spearman’s correlation index *r* and *P*-values are shown on the graphs; the connecting lines illustrate positive and negative correlation indices (**c**)

An increase in the relative weight of the liver was observed at 9 months in animals infected with the PP75 strain via the IC route compared to the other groups. This increase was also observed in the IC group of animals infected with the OP46 strain beginning in the sixth month and persisting at 9 months (Fig. 2a). Macroscopically, the livers of animals infected with both strains and by the IC route appeared friable; changed color from red to pale yellowish brown (Fig. 2b). In hamsters infected with the PP75 strain, the correlation analysis revealed that relative liver weight was negatively correlated with the parasite load in the IP group, but positively correlated in the IC group. In animals inoculated with the OP46 strain, the correlation analysis revealed that relative liver weight was positively correlated with its parasite load in both the IP and IC groups (Fig. 2c).

**Fig. 3:**
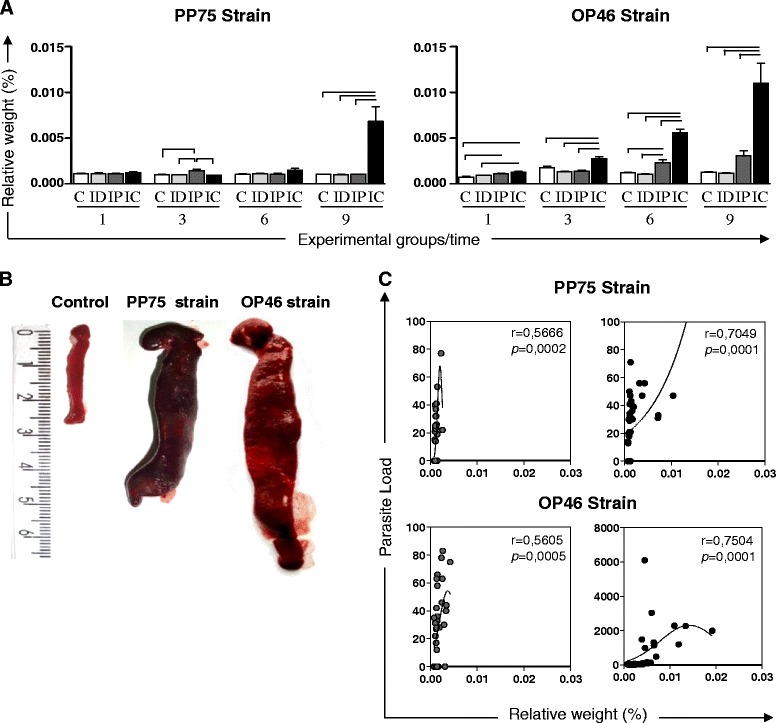
Relative weight of the spleen in uninfected hamsters as a control group (C, *n* = 10 animals/time; white) and hamsters experimentally infected with *L. infantum* (PP75 or OP46 strain) by routes of inoculation: intradermal (ID, *n* = 10 animals/time; light gray), intraperitoneal (IP, *n* = 10 animals/time; dark gray), or intracardiac (IC, *n* = 10 animals/time; black) after 1, 3, 6 and 9 months of infection. The results are expressed as mean ± standard deviation. Significant differences (*P* < 0.05) between infection with the different routes of inoculation are represented by the connected lines (**a**). Macroscopic changes in the spleen of uninfected hamsters as a control group (C) and in hamsters experimentally infected with *L. infantum* (PP75 or OP46 strain) by the IC route of inoculation at 9 months of infection. The macroscopic splenic changes included increase in the volume of the organ (splenomegaly), thickening of edges, rugose and irregular surface and dark coloration (**b**). Correlation between parasite load and relative weight in the spleen of hamsters experimentally infected with *L. infantum* (PP75 or OP46 strain) by different routes of inoculation: intraperitoneal (IP; dark gray) or intracardiac (IC; black) after 1, 3, 6 and 9 months of infection. Spearman’s correlation index *r* and *P*-values are shown on the graphs; the connecting lines illustrate positive and negative correlation indices (**c**)

In the analyses of the spleen, hamsters infected with the PP75 strain and inoculated by the IP route had a slight increase in the splenic relative weight compared with the other groups (C, ID and IC) at 3 months. A greater increase in the splenic relative weight was observed at 9 months after infection by the IC route compared to the C, ID, and IP groups. Animals infected with the OP46 strain via the IP route showed an increase in the relative weight of spleen at 1 month compared to the C group and at 6 months compared to the C and ID groups. The IC route demonstrated a gradual increase in the relative weight of the spleen during the course of infection. This increase was observed with regard to the C, ID, and IP groups at 3, 6, and 9 months, and also in relation to the C and ID groups at 1 month (Fig. 3a). The macroscopic splenic changes most frequently observed included an increase in the volume of the organ (splenomegaly), thickening of edges, rugose and an irregular surface and dark coloration, suggesting hyperemia. It is noteworthy that these alterations were more intense in animals infected with the OP46 strain (Fig. 3b). Furthermore, the correlation analysis revealed that splenic relative weight was positively correlated with the parasite load in the IP and IC groups for both strains (Fig. 3c).

### Hamsters infected with the OP46 strain through the intracardiac route exhibit severe anemia with a reduction of red blood cells, hemoglobin, and hematocrit

In hamsters infected with the PP75 strain, a reduction in the hematocrit was observed in all groups (ID, IP, and IC) after 1 month of infection. At 3 months after infection, an increase in the number of platelets was observed in the IP and IC groups compared to the control animals. Reduced counts for erythrocytes, hemoglobin and hematocrit were observed at 6 months. The only difference at 9 months, however, was an increase in the number of erythrocytes in the IP group compared to the C and ID groups.

In animals infected with the OP46 strain, severe anemia was observed in the IC group from the sixth month of infection, with a reduction of red blood cells, hemoglobin and hematocrit in comparison with the other groups (Table 2).

**Table 2:** Erythrogram of control hamsters and hamsters experimentally infected with strains PP75 and OP46 of *L. infantum*

Hematological variables by group	Time/months							
	1	3	6	9	1	3	6	9
C group								
Erythrocytes (10^6^/mm^3^)	7.2 ± 0.3	7.2 ± 1.1	8.5 ± 1.2	8.4 ± 1.0	7.4 ± 0.4	7.0 ± 0.3	7.1 ± 0.2	6.7 ± 0.3
Hemoglobin (g/dL)	13.9 ± 0.6	13.5 ± 0.8	17.6 ± 2.1	17.5 ± 2.3	15.0 ± 1.0	13.3 ± 0.9	13.3 ± 0.5	13.2 ± 0.7
Hematocrit (%)	35.5 ± 3.6	34.6 ± 7.2	39.5 ± 5.5	38.4 ± 6.7	32.6 ± 1.5	30.5 ± 2.3	32.5 ± 2.3	33.2 ± 2.0
Platelets (10^6^/mm^3^)	165.8 ± 37.4	142.6 ± 33.8	154.9 ± 143	127.4 ± 12.5	139.9 ± 20.8	136.4 ± 16.9	164.7 ± 15.5	174.0 ± 20.5
	Strain/months after infection							
	PP75 strain				OP46 strain			
	1	3	6	9	1	3	6	9
ID group								
Erythrocytes (10^6^/mm^3^)	6.5 ± 0.4	7.2 ± 0.2	6.5 ± 0.4^a^	8.5 ± 0.7	7.4 ± 0.3	6.8 ± 0.3	6.7 ± 0.8	6.9 ± 0.3
Hemoglobin (g/dL)	13.7 ± 1.1	13.6 ± 0.5	12.7 ± 1.0^a^	19.7 ± 4.5	15.2 ± 0.6	13.3 ± 0.7	12.6 ± 1.6	13.7 ± 0.3
Hematocrit (%)	29.3 ± 2.1^a^	36.3 ± 3.0	31.1 ± 2.7^a^	38.3 ± 3.9	33.9 ± 1.5	31.2 ± 3.0	33.6 ± 3.6	34.0 ± 2.0
Platelets (10^6^/mm^3^)	123.8 ± 16.2	168.70 ± 25.26	138.3 ± 19.2	108.1 ± 18.0	156.8 ± 27.5	141.1 ± 24.4	185.5 ± 24.4	189.4 ± 27.3
IP group								
Erythrocytes (10^6^/mm^3^)	6.1 ± 0.1	6.8 ± 0.7	6.7 ± 0.2^a^	9.8 ± 0.5^a,b^	7.5 ± 1.0	7.1 ± 0.4	7.0 ± 0.5	6.2 ± 1.0
Hemoglobin (g/dL)	13.4 ± 0.5	13.1 ± 1.2	13.4 ± 0.4^a^	21.2 ± 1.3	14.4 ± 0.6	13.6 ± 1.2	12.8 ± 1.1	12.0 ± 2.0
Hematocrit (%)	28.9 ± 1.5^a^	38.7 ± 3.2	31.2 ± 2.7^a^	43.9 ± 2.2	32.0 ± 2.6	31.4 ± 3.2	36.1 ± 2.6	31.0 ± 4.2
Platelets (10^6^/mm^3^)	134.1 ± 45.3	221.6 ± 75.0^a^	132.2 ± 23.4	126.3 ± 14.5	169.3 ± 35.4	145.6 ± 32.1	184.4 ± 32.7	174.6 ± 87.4
IC group								
Erythrocytes (10^6^/mm^3^)	6.6 ± 0.4	7.2 ± 0.7	6.8 ± 0.3^a^	9.2 ± 1.0	7.1 ± 0.4	6.7 ± 0.3	4.3 ± 1.4^a,b,c^	4.7 ± 1.9^a,b, c^
Hemoglobin (g/dL)	13.7 ± 0.8	13.8 ± 1.2	13.6 ± 0.7^a^	20.0 ± 2.4	14.5 ± 0.9	12.3 ± 1.6	7.0 ± 1.8^a,b,c^	10.1 ± 4.2^a,b^
Hematocrit (%)	30.1 ± 1.8^a^	35.0 ± 3.6	31.5 ± 2.4^a^	41.5 ± 4.3	31.6 ± 2.1	29.0 ± 4.4	21.0 ± 6.9^a,b,c^	25.0 ± 9.4^a,b^
Platelets (10^6^/mm^3^)	134.1 ± 21.5	144.6 ± 22.3^c^	135.4 ± 13.5	108.5 ± 17.5	156.5 ± 17.0	110.9 ± 29.8	329.4 ± 153.5^a,b,c^	291.3 ± 136.4^a,b,c^

Values (mean ± standard deviation) erytrogram of uninfected hamsters as a control group (C) and infected animals with strains PP75 and OP46 of *L. infantum* by different routes of inoculation: intradermal (ID), intraperitoneal (IP), intracardiac (IC). Significant differences (*P* < 0.05) are represented by letters ^a, b, c^related to the C, ID and IP groups respectively

With regard to the white blood cells, data analysis revealed statistical differences in the animals infected with the PP75 strain at 3 months, with a reduction in the number of eosinophils in animals inoculated by the IP route in relation to the C group. Moreover, at 9 months a significant decrease in the monocyte population was observed in the IC group compared with the control group. In contrast, after 1 month of infection with the OP46 strain by the ID and IP routes, there was an increased number of monocytes in relation to the C group. After 3 months of infection an increase in the lymphocyte population was observed in the group inoculated via the IP route compared to group C. At this same time, animals infected via the IC route presented with leucopenia compared to the C, ID, and IP groups, which was characterized by a remarkable reduction in the monocyte population compared to the C group. After 6 months of infection, the IC group showed some alterations: a reduction in the total number of neutrophils was observed compared to the ID group; the number of eosinophils in relation to the C and ID groups was diminished; the number of lymphocytes was reduced in relation to the C, ID, and IP groups; and the number of monocytes declined in relation to the C and IP groups. In a similar manner, at 9 months after infection with the OP46 strain, hamsters inoculated by the IP and IC routes were more likely to have leucopenia compared to the C and ID groups, which was characterized by a decrease in the total neutrophils, eosinophils, and monocytes (data not shown).

### Hamsters demonstrated consistent biochemical changes which present themselves as strong clinical markers of visceral leishmaniasis

**Fig. 4:**
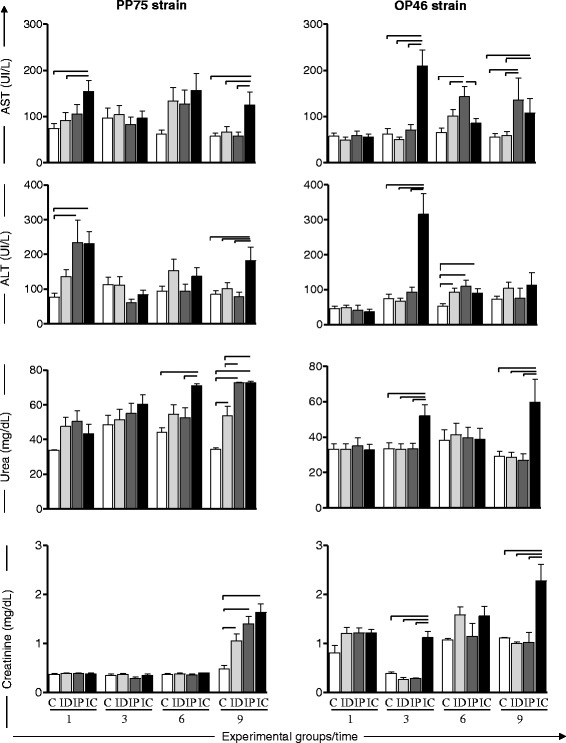
Activity of AST and ALT; urea and creatinine in the blood plasma of uninfected hamsters as a control group (C, *n* = 10 animals/time; white) and in hamsters experimentally infected with *L. infantum* (PP75 or OP46 strain) by routes of inoculation: intradermal (ID, *n* = 10 animals/time; light gray), intraperitoneal (IP, *n* = 10 animals/time; dark gray), or intracardiac (IC, *n* = 10 animals/time; black) after 1, 3, 6 and 9 months of infection. The results are expressed as mean ± standard deviation. Significant differences with *P* < 0.05 between infection with the routes of inoculation are represented by the connected line

The biochemical evaluations are shown in Fig. 4 and consist of the following tests: liver function tests, including the measurement of AST and ALT, and renal function tests based on urea and creatinine levels.

In animals infected with the PP75 strain, an increase in the serum AST level was observed in the IC group in relation to the C and ID groups at 1 month as well as at 9 months in comparison to all other groups (C, ID, and IP). Furthermore, an increase in the serum ALT level was observed in the IC group at 1 month compared to the C group and at 9 months in relation to all other groups (C, ID, and IP) (Fig. 4).

In hamsters infected with the OP46 strain, an increase in the serum AST level was observed in the IC group compared to all groups at 3 months, and in relation to the C and ID groups at 9 months after infection. Similarly, the IP group showed an increase in the AST level in relation to all groups at 3 months after infection and at 9 months in relation to the C and ID groups. Serum ALT levels were observed to be increased in the IC group compared to all groups at 3 months. Furthermore, an increase of ALT was observed in the ID, IP, and IC groups compared with the control group at 6 months of follow-up (Fig. 4).

Regarding renal function, increased levels of urea were observed in the IC group compared to the C and IP groups at 6 months after infection with the PP75 strain. Moreover, after 9 months of infection, increased levels of urea were observed in the three experimental groups (ID, IP, and IC) compared to C group. Also, at the last follow up, an increased serum level of urea in the IP and IC groups in relation to the ID group was detected. In the animals infected with the OP46 strain, increased levels of urea were observed in the IC group compared to all other groups (C, ID, and IP) at 3 and 9 months (Fig. 4). The animals infected with the PP75 strain presented increased serum creatinine levels in all infected groups (ID, IP, and IC) compared to the control group at 9 months (Fig. 4). Furthermore, animals infected with the OP46 strain also presented an increased serum creatinine level in the IC group compared to the others (C, ID and IP) at 3 and 9 months after infection (Fig. 4).

## Discussion

In this study we investigated the clinical and laboratory changes in male golden hamsters infected with *L. infantum* in order to improve our understanding of the pathogenesis studies and clinical aspects of VL. Previous studies by Moreira et al. [7] demonstrated progressive disease in hamsters infected with *L. infantum*, which was closely related to the presence of high parasitism in the spleen and liver. Herein we evaluated the clinical signs and changes in hematological and biochemical parameters in hamsters that were experimentally infected by routes (ID, IP, and IC) with strains of *L. infantum* (MHOM/BR/74/PP75 and MCAN/BR/2008/OP46) that exhibit distinct degrees of virulence and pathogenicity.

The clinical signs observed in our study have also been reported by several other researchers [14, 23, 37–39]. Wilson et al. [40] documented that the clinical symptoms of VL in hamsters only occurred 10 months after ID inoculation of *L. donovani*. However, other studies found that progressive VL was achieved after intradermal infection with 10^5^ parasites, which is the route closer to natural transmission by sand fly bites [22]. The hamsters showed clinical signs similar to those observed in symptomatic HVL, including splenomegaly and severe weight loss. In contrast, the frequency of clinical signs when the ID route was used with both strains was relatively low during our study. However, clinical signs were more evident at 9 months after infection with the PP75 strain inoculated by the IC route, but with low frequency. With the OP46 strain, the clinical signs were more frequent and were exacerbated in the IC group. In other words, our results demonstrated an increased pathogenicity of this strain.

At 9 months after infection with the OP46 strain by the IC route, an accentuated weight loss (cachexia) was observed, as well as mucocutaneous lesions accompanied by localized ulcers in the snout and edema in the paws. We speculate that the cachexia may have arisen due to parasites spreading to the liver and spleen [7] as well as the secondary mucocutaneous lesions. Moreover, these data emphasize the capacity of the IC route to cause a severe injury in hamsters, including cutaneous lesion and edema of the lower limbs. These data corroborate those reported by Nieto et al. [23] in hamsters infected with promastigotes of *L. infantum* by the IC route. This result clearly demonstrates that classical clinical signs observed and reported in human and canine VL also occur in experimental model hamsters. Therefore, we can infer that the route of the inoculum and the strain used are vital conditions for generating progressive VL in the hamster model.

The classical hepatomegaly was an uncommon finding in hamsters infected with either strains used in the present work. However, we reported an increased relative liver weight in both groups (PP75 and OP46 strain) when infection occurred by the IC route, and the weight was closely correlated with an increased parasite load. Our results indicate hepatomegaly not from the increased size of the liver, but with regard to the weight loss. Hepatomegaly is not necessarily always observed during the disease, which has also been documented by other authors [41, 42]. These differences, as well as the other changes, are probably inherent to the complexity of the effects caused by the parasite and the host immune response. Macroscopically, the liver of animals inoculated via the IC route appeared friable; containing white foci distributed across the liver surface and changed color from red to pale yellowish brown. These same macroscopic changes were also described by other authors for hamsters experimentally infected with *L. infantum* and *L. donovani* using the IC route for the inoculums [42, 43]. At 9 months, the spleen size in animals inoculated via the IC route was exorbitant compared to the control group for both strains. We hypothesized that this splenomegaly was due to the high parasitism displayed by these animals [7].

Knowledge of the biochemical and hematological parameters in hamsters experimentally infected with *L. infantum* is extremely limited. Although hematological and biochemical profiles have limited applications for a VL diagnosis, they can be very important in evaluating the clinical prognosis status, as well as in elucidating the pathogenesis to define therapeutic approaches [8, 44–47]. Blood abnormalities are frequently observed in both humans and animal models for VL [48–50]. Leucopenia, neutropenia, and eosiponenia are frequently found in HVL [51–53]. According to Reis et al. [44] the impaired hematological picture in CVL is associated with severe clinical manifestations and demonstrated by leucopenia, which is characterized by lymphopenia, eosinopenia and monocytopenia. Herein, the changes in the hematological parameters of hamsters infected with the PP75 strain were not expressive; we detected monocytopenia in the last month of infection only in the IC group. However, the hamsters infected with the OP46 strain through the IC and IP routes presented intense monocytopenia accompanied by leucopenia after the third month of infection. The leucopenia observed in the IC and IP groups at 6 and 9 months after experimental infection, showed a typical LV immunosuppression marked by a decrease of neutrophils, eosinophils, lymphocytes and/or monocytes. Thus, in the context of our study, the classical hematological changes observed in human and canine VL have been reproduced. The results also confirm that the hematological changes in the IP and IC groups, accompanied by the presence of clinical signs suggestive of active VL (especially splenomegaly), are associated with high tissue parasitism (spleen and liver) previously observed by Moreira et al. [7]. These findings resulted from the complex interaction related to the potential virulence and pathogenicity of each of the two strains used.

Normocytic normochromic anemia is a common finding in patients with the chronic form of the human disease [52] and also for dogs with CVL [44]. In our study the main changes in the red blood cells of hamsters infected with the PP75 strain occurred at 1 and 6 months after infection in the three experimental groups (ID, IP, and IC). Moreover, in animals infected with the OP46 strain, these changes were observed from the sixth month, and were restricted to the IC group only, in which severe anemia, marked by the reduction of erythrocytes, hematocrit and hemoglobin was observed. Anemia was also demonstrated by Rica-Capela et al. [38] in 70–80 % of hamsters experimentally infected with different forms (promastigotes and amastigotes) of *L. infantum*.

Changes in the levels of serum aminotransferase enzymes (ALT and AST) are considered important indicators of hepatocyte structural damage. Measuring serum levels of ALT and AST allowed the presence of changes in the permeability of these cells to be determined. Although a few studies have attempted to assess alterations in the levels of AST and ALT in the LV hamster model, several authors have reported increased serum levels of these enzymes in HVL [47] as well as in CVL [54–56]. Thus, we believe that laboratory monitoring of AST and ALT should be considered when using this model mainly for drug studies for VL. Herein, the increased ALT and AST levels in hamsters from the IP and IC groups at different time points were observed for both strains (PP75 and OP46). Our results provide advanced understanding of the role of these biomarkers in the hepatic function during ongoing experimental hamster infection.

Evaluation of renal function consisted of determining serum levels of urea and creatinine. Our results revealed a renal alteration characterized by an increase in urea and creatinine in both strains by different routes of inoculation and at different time points. The most common change in renal physiology in VL is glomerulonephritis, while amyloidosis is less common [57]. Studies conducted by Rica-Capela et al. [38] showed a large amount of amyloid in the liver and spleen of hamsters experimentally infected with amastigotes and promastigotes of *L. infantum*. These data suggest kidney injury in the animals, perhaps due to the deposition of immune complexes in lymphoid organs.

Therefore, these findings represent an advance in the knowledge of the involvement of experimental infection in hamster model with *L. infantum*. Data indicate that the infectivity capacity of the parasite, the inoculum routes and the dose used as well as the biological form of the parasite (amastigote and promastigote) can influence the course of the natural history of experimental VL. These aspects make it difficult to select an ideal experimental model that is capable of reproducing the disease in its visceral form exhibiting various clinical manifestations and evolving from acute to chronic form [7, 14, 21]. However, here we showed that the route of infection and the strain used was crucial to influence the biochemical and hematological parameters evaluated in this study. Thus the IC route proved to be more aggressive than other routes used and OP46 more virulent and a more pathogenic strain compared to the reference PP75 strain.

## Conclusion

Taken together, the results obtained from this experimental infection of male golden hamsters infected with *L. infantum* having distinct virulence and pathogenicity profiles show that it is possible to reproduce a disease with differing degrees of severity. Many alterations and typical biomarkers, such as hematological/biochemical factors and pathological and clinical signs frequently observed in human and/or canine VL, were also detected in this hamster model. Thus, the results presented in this paper indicate that the golden hamster (*M. auratus*) constitutes an excellent model for analysis of experimental infections caused by different strains of *L. infantum* inoculated by different routes, reinforcing its use in pathogenesis studies of VL.
